# Exercise training attenuates diabetes-induced cardiac injury through increasing miR-133a and improving pro-apoptosis/anti-apoptosis balance in ovariectomized rats

**DOI:** 10.22038/IJBMS.2019.36731.8750

**Published:** 2020-01

**Authors:** Parisa Habibi, Alireza Alihemmati, Nasser Ahmadiasl, Abolfazl Fateh, Enayat Anvari

**Affiliations:** 1 Neurophysiology Research Center, Hamadan University of Medical Sciences, Hamadan, Iran; 2 Department of Histology & Embryology, Tabriz University of Medical Sciences, Tabriz, Iran; 3 Drug Applied Research Center, Tabriz University of Medical Sciences, Tabriz, Iran; 4 Department of Mycobacteriology and Pulmonary Research, Pasteur Institute of Iran, Tehran, Iran; 5 Department of Physiology, School of Medicine, Ilam University of Medical Science, Ilam, Iran

**Keywords:** Apoptosis regulatory-proteins, Caspase-3, Caspase-8, Diabetes mellitus, MicroRNAs, Type 2

## Abstract

**Objective(s):**

The useful and effective role of exercise program to prevent cardiac tissue apoptosis and fibrosis in ovariectomized type 2 diabetic (T2DM) rats (OVR.D) is well known. The current study aimed to investigate the simultaneous effects of T2DM and swimming plan on the expression of some apoptotic, anti-apoptotic biomarkers and glycogen changes in the cardiac muscle tissue of ovariectomized (OVR) rats.

**Materials and Methods:**

Forty rats were randomly sorted into 4 equal categories; sham, OVR, OVR.D and diabetic ovariectomized with an 8 week of swimming plan (OVR.D.E). Lipid profile and miR-133, *Bcl-2*, Bax, caspase-3 and caspase-8 levels were evaluated in the cardiac tissue.

**Results:**

Ovariectomy significantly (*P-value<*0.05) increased cholesterol, triglyceride, LDL, Bax, caspase-3, caspase-8 and decreased (*P-value<*0.05) HDL, miR-133, *Bcl-2* in the cardiac tissue and a further reduction in the expression of miR-133, *Bcl-2* and an enhancement in Bax, caspase-3 and caspase-8 in OVR.D rats was observed (*P-value<*0.01). However, exercise training significantly reversed all the measured parameters (*P-value<*0.05). Also, exercise training improved abnormal tissue structure, fragmentation and irregular form of glycogen granules in the OVR.D.E compared to OVR and OVR.D animals.

**Conclusion:**

Exercise training could prevent the cardiac disturbance, enhance the expression of anti-apoptotic markers and decrease apoptotic biomarkers in the hearts of OVR.D animals. Therefore, based on the findings of this study suggested using the exercise’s beneficial effects for prevention of the cardiac cell death in OVR.D animals.

## Introduction

Diabetes remarkably enhances the cardiovascular disturbances in pre and postmenopausal women (1). Cardiomyopathy is a prevalent adverse effect of diabetes with high morbidity and mortality (2). Estrogen deprivation also induces cardiac impairment in young and postmenopausal females, thus increasing cardiovascular risk (3). Exercise training is a well-known protective strategy used in human and animal models to overcome the cardiac-injurious effects following estrogen insufficiency caused by diabetes (4-6). Also, it has various positive effects such as avoiding diabetes succession by improving insulin sensation, β-cell function, lipid profile and cardiac function (5).

Studies on microRNAs established the role of these molecules in different biological functions including cell growth, cellular differentiation and proliferation, metabolism, survival and apoptotic process. In addition, miRs are suggested as potential biological markers and/or therapeutic goals in several diseases including obesity, diabetes and cardiovascular disorders (7). MiR-133 is mostly expressed in myocytes and fibroblasts in cardiac muscle tissue. Overexpression, targeted deletion, or knockdown of miR-133 genes via antisense targeting discloses its different roles in myocardial remodeling (8, 9). Some important issues are highlighted regarding the participation of miRs such as miR-133 in gene expression regulation after menopause (10) or diabetes (11, 12). Deregulation of microRNA involved in the pathogenesis processes mediating diabetic cardiomyopathy, apoptosis, as well as cell survival (13). Upstream of Bcl-2 protein controls the cell death mitochondrial signaling pathway and microRNAs control its gene expression (14). Some research has shown that miR*-*133a suppresses the expression of several apoptosis regulating proteins including caspase proteins (caspase-3, 8 and 9) and improves the *Bcl-2 *gene expression (15). The authors’ previous study showed alteration in miR*-*133 expression and the effect of exercise on this factor in the cardiac muscle tissue of ovariectomized (OVR) animals (16). Nevertheless, the role of miR-133 in the cardiac muscle of diabetic ovariectomized with 8 week of swimming plan (OVR.D.E) rat is not reported. The current study aimed to investigate whether myocardial-specific miR-133a was involved in the protective effects of exercise plan on estrogen deficiency and diabetes-induced myocardial injury by regulating pro-apoptotic and anti-apoptotic biomarkers.

## Materials and Methods

***Animals and care***

Forty female Wistar rats (200±20 g, aged ten weeks) were purchased from the Animal Facility of the Tabriz University of Medical Science (TUMS, Tabriz, Iran). All animals were treated under standard conditions (22-24 ^°^C), a 12 hr light-dark cycle, and free access to feed and tap water. In this study, the animals were employed in accordance with the instructions of the Ethics Committee from TUMS (17). The animals were randomly distributed into 4 categories (n=10, in each category); sham operated as well as three OVR groups, namely OVR, ovariectomized type 2 diabetic (T2DM) rats (OVR.D), and OVR.D.E. All animals were deeply anesthetized using of ketamine and xylazine (50 and 10 mg/kg, intraperitoneally respectively). The ovarian tissues were removed and oviducts remained intact with minor damage to the adjacent ovarian tissue (10). Induction of type-2 diabetes achieved by combination of high fat eating plan (HFD), low-dose streptozotocin (STZ, single dose) during one month period. All the trained rats were simultaneously submitted to a swimming training protocol (1 hr for 6 days a week) for an eight-week course. On the 32^nd^ and 57^th^ days, blood samples were collected and used to confirm diabetes and measure biochemical parameters. In addition, cardiac muscle tissue was used for miR-133 and Bcl-2 protein expression and histopathological assessment.

***Induction of type 2 diabetes***

The OVR.D and OVR.D.E rats were fed with high fat eating plan (fat=58 %, carbohydrate=17 %, and protein=25 %) *ad libitum* during one month, and then a single dose (35 mg/kg) of STZ solved in citrate buffer (0.1 Molar, pH=4.5) was injected into the peritoneum. Plasma glucose concentration was assessed after 48 hr from the STZ injection and at the end of experiment with a glucose meter. High fasting blood glucose (FBS˃200 mg/dl) was considered as diabetes (inclusion criteria) (4).

***Exercise training protocol***

The OVR.D.E rats were familiarized with the swimming pool for 5-20 min per day on five consecutive days. Then, they underwent an 8 week swimming program during 6 consecutive days (for 60 min/day). The OVR.D.E rats were assessed 24 hr after their last swimming practice. This kind of swimming was a moderate aerobic exercise previously used and was effective in promoting cardiovascular adaptation (18). 

***Biochemistry analysis***

After blood collection from the retro-orbital venous sinus, the blood glucose level was checked by using a glucose meter (Accu-Chek Active glucometer). Plasma insulin and lipid profile concentration were determined by using ELISA and commercial diagnostic kits (Millipore-USA, Randox–UK, respectively).

***RNA extraction and the cDNA synthesis***

MicroRNA was extracted from the heart tissue using the ParsGenome›s miRNA amplification Kit (ParsGenome, Iran). Nucleic acid quantification (A 260/280 ratio) was assessed by a nanodrop 1000 (Thermo Fisher Scientific, MA, USA) and electrophoresis in agarose gel (3%) stained with GelRed (Biotium, Hayward, CA) appraised the sample integrity. The gene expression of miR-133a was quantitatively measured by real-time polymerase chain reaction (RT-PCR). Table 1 shows sequence bases of the primers in respective genes. The housekeeping gene (miR-191) as internal control method of normalization was chosen for microRNA samples. The cDNA was synthesized using the ParsGenome’s miRNA amplification Kit (ParsGenome, Iran).

***Quantitative real-time PCR***

Briefly, 12.5 μl SYBR Premix, 1 μl forward and reverse primer, 8.5 μl water and 2 μl cDNA as template were used in a final volume of 25 µl. By using a negative control (external control) during the PCR assay was checked the accuracy of amplifications. All the reactions were done on a Rotor-Gene Q real-time PCR instrument. The amplification reactions were thermally cycled as follows: initial denaturation at 95 ^°^C for 10 min, followed by a three-step amplification planning (95 ^°^C for 15 sec), then followed by 60 ^°^C for 30 sec repeated 40 cycles for miR-133a and melting curve analysis. In this study, duplicated runs were done. Quantification of real-time PCR was considered as a rise in the intensity of a fluorescent signal created by SYBR Green dye and its reaction with double-stranded DNA. Changes in gene expression were done by 2^–(ΔΔCT)^ procedure. The primer pair specificity was confirmed by analysis of melting curve following gel staining with GelRed (Hayward, California, USA). 

***Western blotting assay***

Western blot assay carried out for measuring of Bcl-2 level. In brief, sample protein electrophoresis was done by using of a spacer and separation gel (4%, 10% respectively), and proteins were transferred onto (90 mA for 2 hr) a PVDF membrane. Then, the membranes were blocked in 3 % skim milk buffer (2 hr) and then incubated overnight with primary antibodies (4 ^°^C) against the Bcl-2 and β-actin (Santa Cruz Biotechnology, USA) on a shaker. After 4x washing with Tris buffer, membranes were incubated with secondary antibody (Santa Cruz Biotechnology, USA) at room temperature (2 hr) and were revealed using the chemiluminescence (ECL) solution. The pictures of protein bands were made by a visualizing machine. Density of the bands in all gels and blots was digitally quantified by densitometric analysis. 

***Enzyme-linked immunosorbent assay (ELISA)***

The concentration of caspase 3 and 8 measured by colorimetric assay kits (CASP3C, CASP8C, Sigma-Aldrich Company USA). Levels of Bcl-2 and Bax were assayed in the heart tissue lysate sample by the ELISA kits (LifeSpan Biosciences, USA). 

***Histology assay***

Heart tissue samples were fixed in neutral formalin (10%), dehydrated by tissue processor apparatus, then washed by xylol and molded in paraffin. Sections (5 μm) were stained by H&E and periodic acid Schiff (PAS) staining and checked using light microscope (Olympus BH-2, Olympus Optical, Tokyo, Japan). The tissue damages were scored as; zero=nil, one=minor (as; focal myocyte injury), two=mild (as; low multifocal degeneration with low level of inflammatory response, disarrangement, disorganization and vacuolation of myocardial fibers), three=moderate (as; multifocal degeneration with moderate inflammatory response, myocardial fibers disorganization and vacuolation) and four=severe (as; extensive multifocal degeneration with diffuse inflammatory process) (19).

***Statistical analysis***

The statistical analyses were conducted using SPSS version 22 software (SPSS. Inc., Chicago, IL, USA). All data from experimental analysis were presented as mean values±standard error mean (SEM). After the analysis of variance by one-way, were compared the mean data using the *post hoc *Tukey’s test. *P*-value<0.05 was determined to be statistically significant.

## Results

***Body weight (BW) and heart weight (HW)***

BW significantly decreased eight weeks after the experiment in OVR.D rats compared with the sham, OVR, and OVR.D.E groups (*P*-value<0.05). In addition, HW and HW/BW ratio were no statistically significant difference among the experimental groups (Table 2).

***Biochemically results***

Table 3 presents some glucose homeostasis parameters. OVR.D rats showed a statistically significant increase in fasting plasma glucose (FBS) and insulin levels compared to sham, OVR, and OVR.D.E animals (*P*-value<0.05). Moreover, exercise training decreased FBS and insulin concentrations in trained animals (OVR.D.E) compared to the OVR.D group (*P*-value<0.05). Also, HbA1c (glycosylated haemoglobin) concentrations showed an enhancement in the OVR.D animals compared to the sham, OVR, and OVR.D.E groups, but not statistically significant.

**Figure 1:**
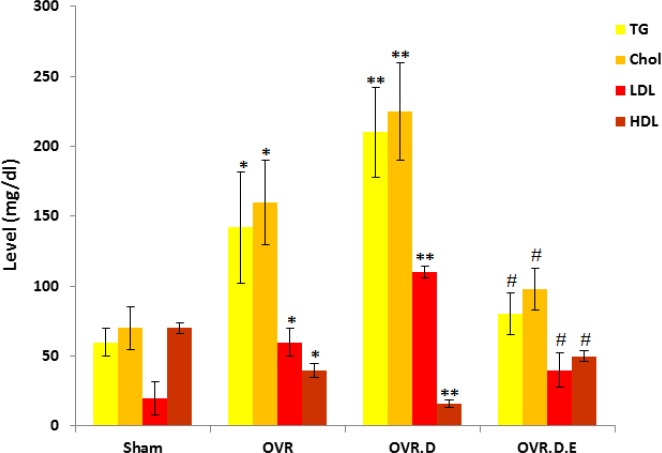
The plasma lipid profile after eight-week intervention (n=10). OVR; ovariectomized animals, OVR.D; ovariectomized with diabetes and OVR.D.E; ovariectomized with diabetes and an eight week swimming program. *Significant difference compared to healthy (sham group, *P-value*<0.05), **compared to sham (*P-value*<0.01) and OVR (*P-value*<0.05), ^#^compared to sham, OVR and OVR.D animals (*P-value*<0.05)

The lipid profile; Cholesterol, TG (triglyceride), and LDL (low-density lipoprotein) levels significantly increased and HDL reduced in the OVR.D group compared to the untrained animals (sham group, *P*-value<0.01). The swimming training declined cholesterol, triglyceride, and LDL and significantly elevated HDL level in the trained animals (OVR.D.E) compared to the OVR and OVR.D animals (*P*-value<0.05) (Figure 1). 

***Expression of MiR-133***

**Figure 2:**
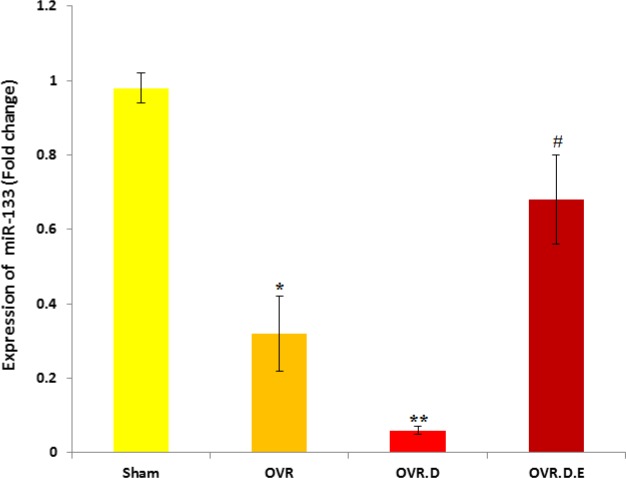
MiR-133 expression levels in the cardiac tissue of four groups of animals (n=10). OVR; ovariectomized animals, OVR.D; ovariectomized with diabetes and OVR.D.E; ovariectomized with diabetes and an eight week swimming program. *Significant difference compared to healthy (sham, *P-value*<0.05), **compared to Sham and OVR animals (*P-value*<0.05), ^#^compared to sham, OVR and OVR.D animals (*P-value*<0.05)

Expression level of MiR-133 as significantly reduced in the cardiac muscle tissue of the OVR and OVR.D groups (*P*-value<0.05) compared to the untrained animals (sham). Swimming training significantly increased that in the cardiac muscle tissue of the trained group (OVR.D.E) compared to the OVR.D (*P*-value<0.05), OVR (*P*-value<0.05) and sham groups (*P*-value<0.05) (Figure 2).

***Gene expression of Bcl-2***

**Figure 3:**
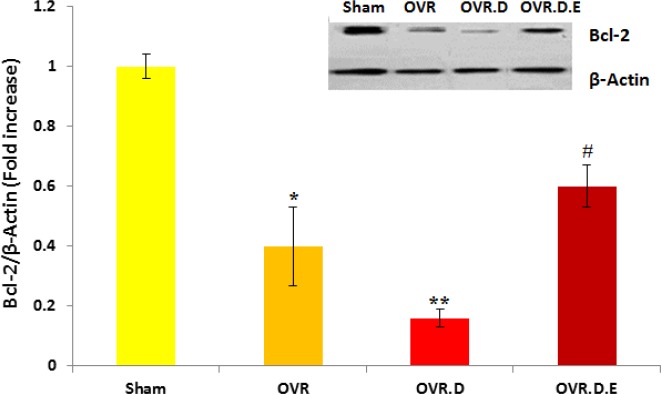
Bcl-2 protein level in the cardiac tissue of four groups of animals (n=10). OVR; ovariectomized animals, OVR.D; ovariectomized with diabetes and OVR.D.E; ovariectomized with diabetes and an eight week swimming program. *Significant difference compared to healthy (sham) animals (*P-value*<0.05), **compared to sham and OVR animals (*P-value*<0.05), ^#^compared to sham, OVR, OVR.D animals (*P-value*<0.05)

The gene expression level of *Bcl*-*2* was reduced significantly in the hearts of the OVR group compared to the untrained animals (sham group, *P*-value<0.05). In addition, the research findings showed a further reduction in the expression level of *Bcl-2* in the cardiac tissue of the OVR.D animals compared to the OVR (*P*-value<0.05), sham animal groups (*P*-value<0.01). Swimming training significantly enhanced the expression level of *Bcl*-*2* in the cardiac tissue of the trained animals compared to the OVR.D animals (*P*-value<0.05) (Figure 3).

***Levels of the apoptotic proteins***

**Figure 4:**
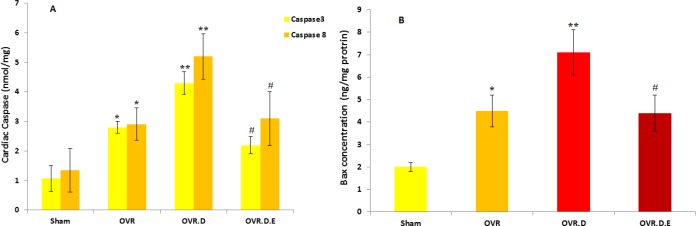
The levels of apoptotic proteins caspase-3 and caspase-8 (A) and Bax (B) in the cardiac tissues of different studied groups (n=10). OVR; ovariectomized animals, OVR.D; ovariectomized with diabetes and OVR.D.E; ovariectomized with diabetes and an eight week swimming program. *Significant difference compared to healthy (sham) animals (*P-value*<0.05), **compared to sham (*P-value*<0.01) and OVR (P-value<0.05), ^#^compared to OVR.D and sham animals (*P-value*<0.05)

The apoptotic proteins levels significantly increased in the cardiac tissue of the OVR category compared to the untrained (sham category,* P*-value*<*0.05) and diabetes animals (*P*-value*<*0.01). However, the apoptotic proteins levels significantly reduced in the cardiac tissue of the trained animals (OVR.D.E, *P*-value*<*0.05) (Figure 4A and B). 

***Histological results***

**Figure 5:**
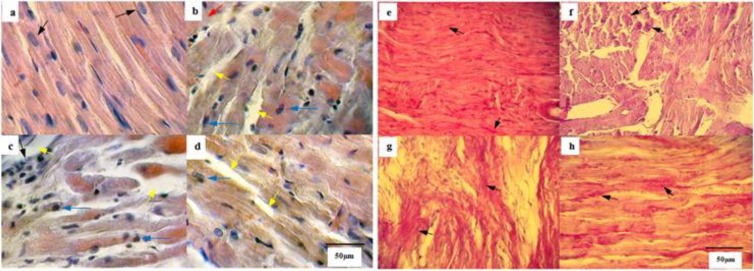
Histological evaluation of myocardium under light microscopy using haematoxylin and eosin (H & E) stain (a, b, c and d) and periodic acid–Schiff (PAS) staining (e, f, g and h). H&E staining of the hearts in healthy (sham, a), OVR (b), OVR.D (c) and OVR.D.E (d). Striped cells of cardiac, oval, vesicular, pale and centrally located nucleus (a, black arrow), acidophilic sarcoplasm (b, red arrow), necrotic cells (b, c, d, blue arrow), tissue fibrosis (b, c, d, yellow arrow), leukocytes infiltration (c, black arrow). PAS staining presented the different storage of glycogen in the cardiac tissue muscle of sham (e), OVR (f), OVR.D (g) and OVR.D.E (h) animals. A homogeneous and uniform expansion of glycogen granules were seen in sarcoplasm (e). Compared to the healthy animals, in OVR and OVR.D groups (f and g) were seen fragmentation and irregular accumulation of glycogen granules (f)

Cardiac tissue histological evaluation showed that swimming training resulted in many significant alterations including fibrosis reduction and a decline in leukocytes infiltration. As shown in Figure 5a, cardiac muscle cells were in normal position with cross-striated appearance. From the view of cell organelles, the nucleus was oval, vesicular, pale and centrally placed. In the OVR group, the sarcoplasm of cardiac myocyte was striped, acidophilic and organized. In addition, we were observed necrotic cells and diffuse fibrosis in this group (Figure 5b). Statistically significant differences were identified in structural integrity among groups. Many of the alterations in the cardiac tissue of the OVR.D group were the same with OVR animals but more severe (Figure 5c). Exercise training decreased the necrotic cells and tissue fibrosis in the OVR.D animals (Figure 5d). Periodic PAS staining analysis showed homogeneous sarcoplasmic granules of glycogen in the cardiomyocyte in the sham group (Figure 5e). Fragmented and irregular granules of glycogen were observed in the OVR and OVR.D animals (Figure 5f), but these were severe in the diabetic ovariectomized category (Figure 5g). Swimming program reduced the breakdown of the glycogen granules in the OVR.D.E compared to the other experimental groups (Figure 5h) (Table 4).

## Discussion

The current study assessed the simultaneous effects of T2DM and swimming program on the expression of miR-133 and some apoptotic biomarkers in the hearts of ovariectomized rats. The major findings of the current study were; 1) diabetes could reduce the expression of miR-133 in the cardiac muscle tissue, probably through a decrease in gene expression of* Bcl*-*2* and an rise in gene expression of* Bax, *caspase 3 and caspase 8; 2) swimming increased the cardiac miR-133 expression probably through enhancement of* Bcl-2* gene expression and anti-apoptotic proteins and decreased Bax, caspase 3 and caspase 8 proteins as apoptotic biomarkers in OVR.D.E animals. 

To date, no data are available on miR-133 and apoptotic and anti-apoptotic biomarkers in the cardiac tissue of an ovariectomy animal model with or without diabetes. The current study was the first to point out an association between cardiac miR-133 and estrogen deficiency-induced cardiac apoptosis and the beneficial effects of exercise on them. 

Some research has mentioned a special role for involvement of miR-133 in cardiac pathogenesis and cardiac cell death pathological remodeling (21, 22) so that miR133 is down-regulated in matrix remodeled, apoptotic, dysfunctional and hypertrophic hearts (6, 12, 20). An explicit link between a myocardial miR-133 down-regulation and a boosted expression of fibrosis markers was reported in an animal model with STZ-induced diabetic cardiomyopathy (21). Moreover, overexpression of miR-133 by transgenic methods reversed cardiomyopathy remodeling by castration of these fibrotic markers (6). In confirmation of the mentioned effect, subjecting a STZ-induced diabetic cardiomyopathy mice model to a 10-week swimming program unregulated the miR-133, improved contractile properties and reduced an extracellular matrix (ECM) regulatory protein; metallopeptidase-9 (MMP_9_) (6). It is also possible that exercise leads to the activation of the myo-miRs in skeletal muscle and their release into the circulation (22). These circulating myo-miRs could reconstruct the depleted myo-miRs in the cardiac tissue. It seems that the cross-talk among skeletal and cardiac muscle is an important molecular mechanism in cardio-protection by exercise (6). In addition, cardiovascular miRs could be affected by exercise in diabetes. For example, low expression of miR-133 was correlated with the enhancement of oxidative stress and dysfunction in cardiac tissue of the diabetic rat (23). However, treating diabetic rats with antioxidants could improve cardiac ultrastructure and heart function (23). Exercise program mediated cardio-protection through modulation of microRNA, could inhibited the enhancement of oxidative stress and some target proteins, in the diabetic animals by several molecular pathways. Hence, exercise training resulted in the suppressing of apoptosis and cardiac remodeling. Does the exercise program play a role in the protection of the diabetic cardiomyopathy through the regulation of miRs? It has not been fully understood yet (6). But it is known that acute resistance and endurance physical activities (exercise) in males were able to promote the miR-133 expression (6).

Chen *et al., *showed that miR-133a overexpression in diabetic mice could prevent the extracellular matrix (ECM) proteins overexpression and focal cardiac fibrosis enhancement that significantly decreased cardiac fibrosis. The protective response by miR133 overexpression includes the reduced ERK1/2 (extracellular signal–regulated kinase) activation and there-by resulted in an alteration of fibrogenic factors (21). Accordingly, miR-133a could also become a special therapeutic target in treatment of diabetic patients (21).

Earlier studies revealed that estrogen deficiency could increase body weight during and after menopause in OVR rats (24). The augment in the body fat mass is an important cause of increased insulin resistance in estrogen insufficient conditions (25). The current study findings were in line with these results. Indeed, eight weeks of exercise training reduced body weight and prevented the hyperglycemia/hyperinsulinemia in swimming training rats. Also, physical activity improved glucose metabolism and lipid profile in ovariectomized diabetic rats compared to other groups.

Based on the above-mentioned studies, this research was planned for checking the effects of regular exercise program on cardiac miR-133, Bcl-2, Bax, caspase-3 and caspase-8 proteins as a protective strategy in diabetic ovariectomized rats. Also, to confirm the exercise’s beneficial effects on cardiac performance, the histological data from the current study revealed a reduction in fibrosis and necrotic cell number and regulation of the accumulation of glycogen granules in the trained animals compared to the OVR and OVR.D groups.

Due to lack of funding, we couldn’t analyze the more groups such as control+ exercise, OVR+ exercise (ovariectomized with exercise training) and a diabetic group for better comparison of results. 

**Table 1:** The primer sequences of genes in this study

Genes	Accession number	Target sequence^a^
miR-133	MIMAT0017124	AGCUGGUAAAAUGGAACCAAAU
miR-191a	MIMAT0000866	CAACGGAAUCCCAAAAGCAGCUG
^a^Sequences got from; www.mirbase.org		

^a ^Sequences got from; www.mirbase.org

**Table 2:** Body and heart weight in four groups of animals

	Sham	OVR	OVR.D	OVR.D.E
Final body weight (g)	261.42 ± 5.60	272.88 ± 2. 80	182.00 ± 3.29^a^	257.88 ± 4.44
HW (mg)	897 ± 23.39	981 ± 23.53	905 ± 37.80	980 ± 37.0
HW/BW (mg/g)	3.53 ± 0.153	3.43 ± 0.122	4.04 ± 0.263	4.00 ± 0.215

Body weight (BW), heart weight (HW) and heart and body weight ratio (HW/BW). OVR; ovariectomized animals, OVR.D; ovariectomized with diabetes and OVR.D.E; ovariectomized with diabetes and an eight week swimming program (^a^ *P-value<*0.05 vs. Sham, OVR, & OVR.D.E.)

**Table 3:** Metabolic parameters in four groups of animals

	FBS (mg/dl)	Serum insulin (µIU/mL)	HbA1c (%)
Sham	98.20 ± 2.13	4.3 ± 1.8	4.380 ± 0.57
OVR	109.00 ± 1.87	13.2 ± 1.1	4.920 ± 0.63
OVR.D	269.00 ± 13.26^a, b, c^	28.9 ± 1.5^e, f^	5.960 ± 0.60
OVR.D.E	160.00 ±10.83^d^	24.9 ± 1.6^g^	4.620 ± 0.67

Fasting blood glucose (FBS), glycosylated haemoglobin (HbA1c), OVR; ovariectomized animals, OVR.D; ovariectomized with diabetes and OVR.D.E; ovariectomized with diabetes and an eight week swimming program (a, d, e, g vs. Sham, b, f vs. OVR, c vs. OVR.D.E, in all cases *P-value* was <0.05)

**Table 4:** Cardiac muscle tissue changes by H&E staining in four groups of animals

Groups	Cardiac pathology scoring					
	0	1	2	3	4	Mean
Sham	10	0	0	0	0	0
OVR	4	5	1	0	0	0.7
OVR.D	0	0	4	3	4	3.3
OVR.D.E	0	2	3	4	1	2.4

OVR; ovariectomized animals, OVR.D; ovariectomized with diabetes and OVR.D.E; ovariectomized with diabetes and an eight week swimming program (n=10 for each groups). The tissue lesions were scored as; 0 = nil, 1 = minimum (as; focal myocyte injury), 2 = mild (as; low multifocal degeneration with low level of inflammatory response, disarrangement, disorganization and vacuolation of myocardial fibers), 3 = moderate (as; multifocal degeneration with moderate inflammatory response, myocardial fibers disorganization and vacuolation) and 4 = severe (as; extensive multifocal degeneration with diffuse inflammatory process)

## Conclusion

Exercise training could prevent cardiac disturbance and enhance the expression of miR-133 and *Bcl-2 *levels and anti-apoptotic markers and decrease Bax, caspase proteins as apoptotic biomarkers in the cardiac muscle tissue of OVR.D rats. These changes are probably one of the most important ways pertaining to the exercise benefits for prevention of cardiovascular disorders. Therefore, based on the findings of this study suggested using the exercise’s beneficial effects for prevention of the cardiac cell death in OVR and OVR.D animals.
